# Modelling breeding strategies against genetic defects in a vulnerable cattle breed

**DOI:** 10.1371/journal.pone.0355541

**Published:** 2026-08-20

**Authors:** Ebba Gustafsson, Anna M. Johansson, Martin Johnsson

**Affiliations:** 1 Department of Animal Biosciences, Swedish University of Agricultural Sciences, Uppsala, Sweden; 2 Beijer Laboratory for Animal Science, Swedish University of Agricultural Sciences, Uppsala, Sweden; Laboratoire de Biologie du Développement de Villefranche-sur-Mer, FRANCE

## Abstract

This paper investigates different selection scenarios to reduce the prevalence of a genetic defect in a vulnerable breed, using simulations of selection against gonadal hypoplasia in Swedish Mountain Cattle as a case study. Gonadal hypoplasia (reduced size of ovaries/testes) that leads to reduced fertility has long been a problem in Swedish Mountain cattle (Fjällko). Despite breeding efforts that have reduced the frequency of the defect, it is still present in the breed. There are two translocations involving chromosomes 6 and 29 associated with desired colour phenotypes that segregates in several Swedish local cattle breeds. One of these is associated with gonadal hypoplasia although there is incomplete penetrance. With modern DNA methods it is possible to detect the carriers of this defect and tackle this old problem, but the key issue is to do this without harming genetic diversity. We performed simulations with allele frequencies and penetrance that have been estimated previously in the breed and different scenarios of marker-assisted selection. The simulated scenarios were: genetic testing of potential bulls prior to selection to exclude carriers or homozygotes for the deleterious allele; genetic testing of bulls used for artificial insemination to exclude carriers or homozygotes for the deleterious allele; and genetic testing of bulls used for artificial insemination but where 1 or 5 carrier sires were allowed to be used. Simulations showed that it is possible to reduce the frequency of the allele causing the genetic defect while maintaining the effective population size. This however comes with a cost of requiring many genetic tests. Based on these results, the most promising approach would be to pre-screen bull calves before selection of sires, maintaining the number of bulls that are used. These results suggest that efforts to genotype and phenotype cattle for validation and testing of the marker in Swedish Mountain cattle is warranted.

## Introduction

**Fig 1:**
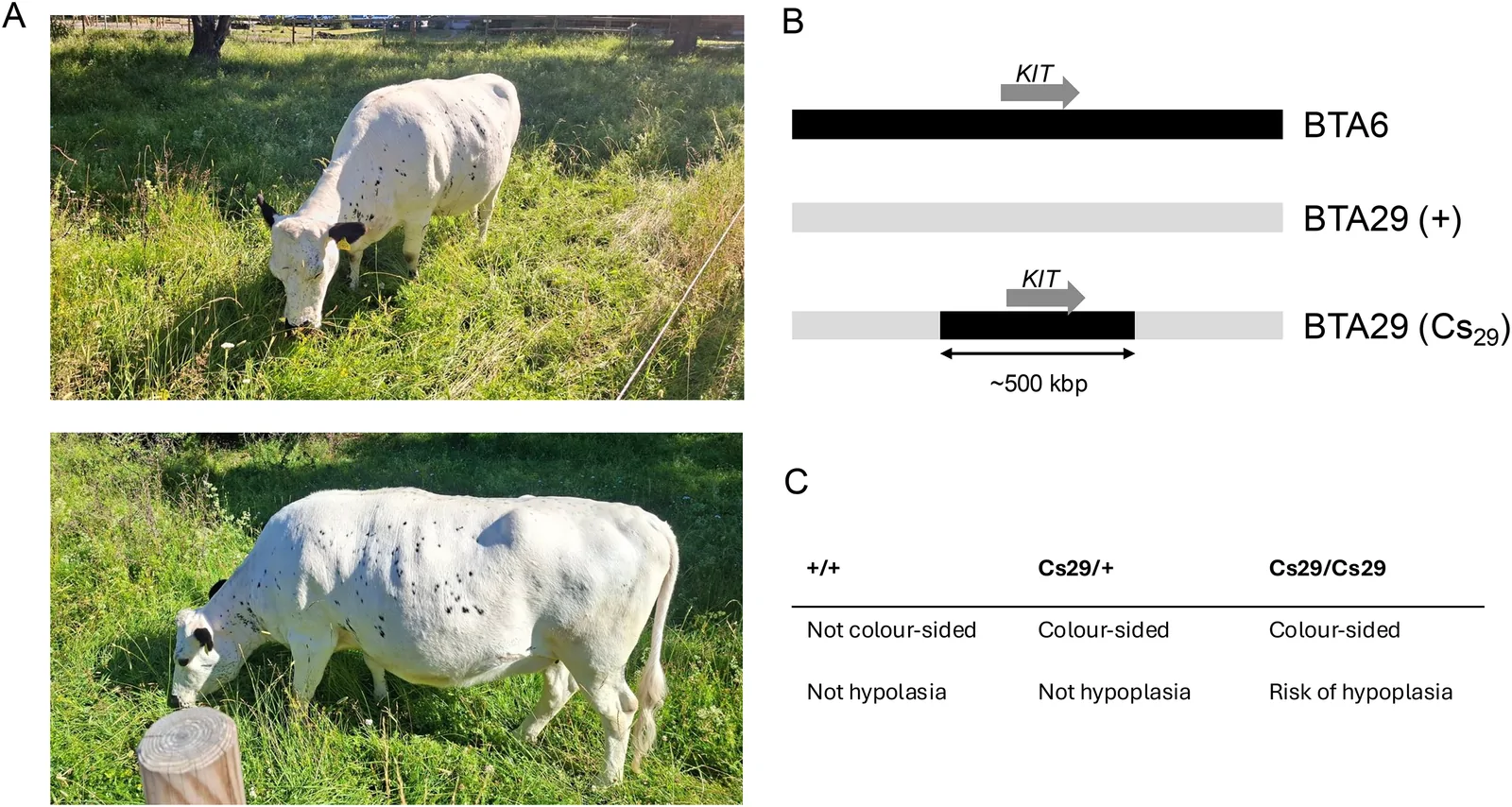
A) A Swedish Mountain cow showing the sidedness phenotype, with characteristic black pigment on the ears and muzzle, white back and mostly white sides with black spotting. B) Schematic image of the Cs_29_ allele, showing how a fragment of around half a megabasepair, including the *KIT* gene have been translocated from chromosome 6 to chromosome 29, contrasted with the wild type chromosome 29. C) Genotype—phenotype matrix describing the associations between Cs_29_ and colour-sidedness and gonadal hypoplasia [1,2,5].

In this work, we analyse the potential for marker-assisted selection against genetic defects without loss of genetic diversity in numerically small, vulnerable breeds, using the gonadal hypoplasia defect in Swedish Mountain cattle as a case study. During the early 20th century, when the Swedish Mountain cattle was still a numerically large breed, there was an increase in a genetic defect that causes gonadal hypoplasia [1]. Gonadal hypoplasia affects both sexes and is characterized by reduced ovarian and testicular size leading to subfertility or even sterility in severe cases. The defects shows an autosomal recessive inheritance pattern and incomplete penetrance, where about half of the homozygotes express hypoplasia, most of them single-sided, usually on the left side. Only a minority develop the severe form of double-sided hypoplasia [1]. The disorder is associated with the Cs_29_ allele consisting of a large translocation between chromosome 6 and chromosome 29, discovered by Durkin et al. to cause colour-sidedness [2]. This is a common coat pattern in Swedish mountain cattle (see Fig 1) and especially the larger white areas on the body of the homozygotes have traditionally been a desired phenotype in the breed [3]. The translocation involves complex rearrangements, hypothesised to happen through circular intermediates. It affects a ~ 500 kbp region including the *KIT* gene, associated with coat colour patterning in many animal species, and results in Cs_29_ carriers having an additional copy of *KIT*, with surrounding region, inserted into chromosome 29, in addition to the normal *KIT* locus on chromosome 6. The association between Cs_29_ and hypoplasia was detected in Northern Finncattle, a Finnish breed which is closely related to Swedish Mountain cattle [4], by Venhoranta et al. [5]. The extremely inbred Chillingham cattle breed, which is colour-sided and where testicular hypoplasia is common, appears to be fixed for Cs_29_ (or have a very high frequency of the allele) [6].

The association with colour-sidedness suggests that the increase of gonadal hypoplasia during the early 20th century was driven by the selection for white coat colour as part of the early breeding goal [7,8]. The combination of a dominant mode of inheritance for colour-sidedness and recessive mode of inheritance for hypoplasia results in heterozygote advantage, where heterozygotes are colour-sidedness but do not suffer the risk of hypoplasia. Thus, gonadal hypoplasia is likely an example of a genetic defect maintained at a high frequency by balancing selection in the form of heterozygote advantage. A phenotypic control program was implemented during the 1940-50s which initially managed to decrease the frequency of the defect [1,9]. Since then, the Swedish Mountain cattle breed has decreased in population size and a key goal for breeding activities has therefore been to maintain the genetic diversity of the breed. The Swedish Mountain cattle is a heritage breed with origin from northern Sweden that is genetically distinct from heritage breeds from southern Sweden and from commercial cattle breeds [10]. A study from a few years ago showed no signs of inbreeding in the breed and that genetic diversity had only decreased a little bit over the last decades [11]. This places the breed in a good position for possibility to breed for reduced frequency of gonadal hypoplasia. The control program for gonadal hypoplasia is still in effect, and the frequency of the Cs_29_ allele is still relatively high within the Swedish Mountain cattle population [12].

It is now possible to genotype the Cs_29_ translocation through molecular methods. Durkin et al. [2] developed breakpoint PCR assays, which were refined by Venhoranta et al. [5], and used by us for multiplex PCR where amplicons for both the wildtype and the Cs_29_ allele are amplified in the same tube [12]. Thus, we set out to explore how breeding strategies could be designed to reduce the frequency of affected animals while simultaneously preserving the genetic diversity of the Swedish Mountain cattle population. If there are realistic prospects for reducing the frequency of gonadal hypoplasia through breeding, the considerable effort to validate Cs_29_ genotyping in Swedish Mountain cattle and making a commercial DNA test available to breeders may be justified. At the time of writing, the Swedish Mountain cattle breed is classified as at risk [13]. Similar situations exist in many livestock and companion animal breeds, where harmful alleles have risen to appreciable frequencies due to genetic drift or balancing selection [14–16]. Marker-assisted selection is an attractive option for reducing the frequency of such defects, since marker-assisted selection works well against monogenic recessive traits that are ineffectively removed by phenotypic selection. However, most animal breeds are effectively small, and loss of genetic diversity is a concern. This is particularly important for vulnerable breeds under conservation efforts.

The project aims to evaluate potential breeding strategies against hereditary gonadal hypoplasia in Swedish Mountain cattle to ascertain how the frequency of the disease-associated allele, and the number of hypoplastic animals can be decreased while maintaining genetic diversity of this local breed at risk. To achieve this, the objectives were to develop a simulation of a deleterious genetic variant under balancing selection in an effectively small population, resembling the Cs_29_ allele associated with gonadal hypoplasia in Swedish Mountain cattle, and use it to simulate potential breeding strategies against gonadal hypoplasia, using phenotypic and genetic testing, and evaluate the effects on the allele frequency of the defect and effective population size.

## Materials and methods

### Study design

The present study utilised stochastic simulations to model and evaluate breeding strategies against a recessive deleterious allele with incomplete penetrance under balancing selection in a population modelled after the Swedish Mountain cattle.

### Modelling

Simulations and subsequent analysis of simulation outcomes were conducted with R version 4.4.2 (2024-10-31 ucrt) [17] using the packages AlphaSimR version 1.6.1 [18], GeneticsPed version 1.68.0 [19] and tidyverse [20].

### Genetic parameters

Haplotypes of the founder population were created with the Markovian Coalescent Simulator software MaCS [21] within AlphaSimR. The historic effective population size was set to 7349, as estimated by Adepoju et al. [22]. The founder generation consisting of 2000 individuals with an equal sex distribution. One chromosome segment was simulated with a physical length of 1 Mbp and a genetic length of 1 cM, to represent the region around the deleterious variant.

The variant representing Cs_29_ was randomly selected from the variants on the first chromosome segment that had an allele frequency of between 0.43 and 0.45. The frequency span was set to encompass the current frequency of the Cs_29_ allele most recently estimated to 0.44 by Hinken et al. [12]. The deleterious allele was treated as pleiotropic with a dominant effect on colour-sidedness and a recessive effect on gonadal hypoplasia. The penetrance of the hypoplasia phenotype was set in accordance with estimates by Eriksson et al. [1] to 0.43 for males and 0.57 for females.

### Breeding structure

To establish a starting point from which alternative strategies could be implemented, selection based on the current management strategy of gonadal hypoplasia in the breed was simulated during the first 20 discrete generations, corresponding roughly to 100 years of selection. In each generation, 90 bulls and a maximum 1000 cows were selected from the available male and female candidates and randomly mated to produce approximately 2000 offspring.

Selection occurred in two steps. First, all animals that expressed the gonadal hypoplastic phenotype were removed from the pool of selection candidates. This resembles the regulation for registration in the herdbook mandating that all animals must be examined and declared free from gonadal hypoplasia to be registered. For simplicity, no restrictions were applied regarding the hypoplasia status of the animal’s dam and maternal granddam, as is otherwise the case in the current control program.

Second, following the removal of affected animals, 90 bulls were selected based on sampling probabilities for the genotypes, determined by their phenotypic expression of coat colour and pattern. These combined effects of the defect result in heterozygote advantage, because heterozygote animals do not express hypoplasia but develop colour-sidedness. This situation is described by the classical deterministic model, reviewed by Hedrick [16]. In this model, the fitness of genotypes is $\text{1}-s_{\text{1}}$ for the wildtype homozygote, 1 for the heterozygote, and $\text{1}-s_{\text{2}}$ for the homozygote for the defect. The expected equilibrium frequency is:


$$\begin{array}{c} q_{eq}=\frac{s_{\text{1}}}{s_{\text{1}}+s_{\text{2}}} \end{array}$$

Where $s_{\text{1}}$ represents the difference in fitness between heterozygote and wildtype homozygote and $s_{\text{2}}$ represents the difference in fitness between the heterozygote and the homozygote for the deleterious allele. The values of both $s_{\text{1}}$ and $s_{\text{2}}$ are uncertain, since the level of subfertility in unilaterally hypoplastic animals and the strength of farmer preference for colour-sidedness are not known. With a control program, the deleterious effect becomes equal to the penetrance, as all hypoplastic animals are excluded $s_{\text{2}}\approx \text{0}.\text{5}$. The inferred allele frequency after the instatement of the control program and the current allele frequency of Cs_29_ are around 44% [9,12]. Under the classical model, this corresponds to a coefficient of positive selection $s_{\text{1}}\approx \text{0}.\text{4}$. However, with sex-specific penetrance, the negative selection due to the control program is somewhat weaker for males, and consequently the positive selection required for equilibrium is somewhat weaker. To achieve a realistic equilibrium frequency in the simulation, we tested different values of the coefficient of positive selection. S1 Fig shows simulations with different values for the selection coefficient $s_{\text{1}}$ and the resulting equilibrium frequency. A selection coefficient of $s_{\text{1}}=\text{0}.\text{3}$ led to a relatively stable allele frequency close to the initial value of 0.44, as can be seen in result of the baseline scenario, and the simulated population therefore appeared to be close to the equilibrium. Increasing or decreasing $s_{\text{1}}$ to 0.20 or 0.40, respectively, led to substantially higher or lower frequencies.

After selection, the 90 bulls were then randomly divided into three groups of 50, 25 and 15 bulls with low, medium and high numbers of offspring. The groups roughly reflect the number stock bulls, AI (artificial insemination) bulls and elite AI sires used in breeding within the Swedish Mountain cattle breed each generation, and the proportions were set to give an effective population size similar to that of the current population. For the low offspring group, representing stock bulls, the number of offspring per sire was sampled from a Poisson distribution with a mean of 1, plus 1 to ensure at least one offspring per sire. Of the remaining offspring 40% were assigned to the medium offspring group representing AI bulls and 60% to the high offspring group representing elite AI sires. The average number of offspring per sire for these groups were then calculated by dividing the respective total number of offspring per group by the number of sires in each group rounding up to the nearest integer. Sires assigned to produce more than one offspring were repeated according to the number of offspring in the final list of selected sires.

Next, 1000 dams were selected from the pool of gonadal hypoplasia-free candidates using the same selection probabilities previously used for the bulls. However, unlike the bulls, individual dams were allowed to be selected more than once. This adjustment was made to account for the possibility that there may be fewer than 1000 gonadal hypoplasia-free females available for selection in some generations. The number of offspring per dam was adjusted to account for variations in the total number of offspring per generation and like the final list of sires, the IDs of dams assigned more than one offspring were repeated in the final list of dams.

Finally, mating of selected sires and dams was simulated. The mating pairs were assigned randomly and there were no restrictions preventing the mating of closely related individuals, such as full or half siblings.

The goal was to obtain an average effective population size of the simulated population in generation 21–40, across ten replicates, close to that of the actual population (estimated by Adepoju et al. [22] to around 136). The total number of bulls selected for breeding and the distribution offspring across the selected bulls were adjusted to achieve an effective population size close to this estimate. The resulting baseline scenario had an effective population size across 40 replicates of 139 ± 16.6.

### Selection scenarios

In total, five alternative selection scenarios aiming to reduce the frequency of both the deleterious allele and the frequency of affected animals were simulated. Initially, baseline selection as described above, was applied across all scenarios during the first 20 generations. Following this burn-in phase, each alternative selection strategy was then implemented from generation 21–40. In scenarios where genetic testing was applied, it was assumed to be completely accurate without genotyping errors. Each scenario was replicated 40 times.

- *Pre-test homozygous excluded*. Genetic testing of bulls prior to selection to exclude those homozygous for the deleterious allele. Under this scenario, including four sub scenarios, 25%, 50%, 75%, and 100% of all unaffected bulls in each generation were selected for genetic testing to identify those homozygous for the deleterious allele. The identified homozygous bulls were subsequently removed from the pool of breeding candidates before bulls were selected.
- *Pre-test carriers excluded*. Genetic testing of bulls prior to selection to exclude carriers of the deleterious allele. The procedure was identical to that described in the previous scenario with genetic testing of varying proportions of unaffected bulls. However, in this third scenario both homozygous bulls and carriers of the deleterious allele were excluded from breeding prior to selection and mating.
- *Post-test AI bulls homozygous excluded*. Genetic testing of all bulls in the medium and high fecundity groups following selection to exclude those homozygous for the deleterious allele from breeding. Under this scenario 90 bulls where selected and divided into fecundity groups as described in the baseline scenario. Following this all, 40 bulls belonging to either the medium or high fecundity group were genetically tested and all individuals homozygous for the deleterious allele were excluded from breeding without replacement. The medium and high fecundity groups still received the same proportion of the total number of offspring as in the baseline scenario. This is a pessimistic scenario, reflecting limited availability of AI bulls in practice.
- *Post-test AI bulls carriers excluded*. Genetic testing of all bulls in the medium and high fecundity groups following selection to exclude carriers of the deleterious allele from breeding. The procedure was equal to the previous scenario, except that both homozygous bulls and carriers of the deleterious allele in the medium and high fecundity groups were excluded from breeding.
- *Post-test AI bulls carriers excluded with exemptions*. This scenario attempted to maintain the effective reduction in frequency of the deleterious allele from removing carrier AI bulls while mitigating the decrease in effective population size. In this scenario, carriers and homozygotes were excluded from breeding as in the previous scenario, but 1 or 5 carrier sires were allowed among the elite AI bulls.

**Fig 2:**
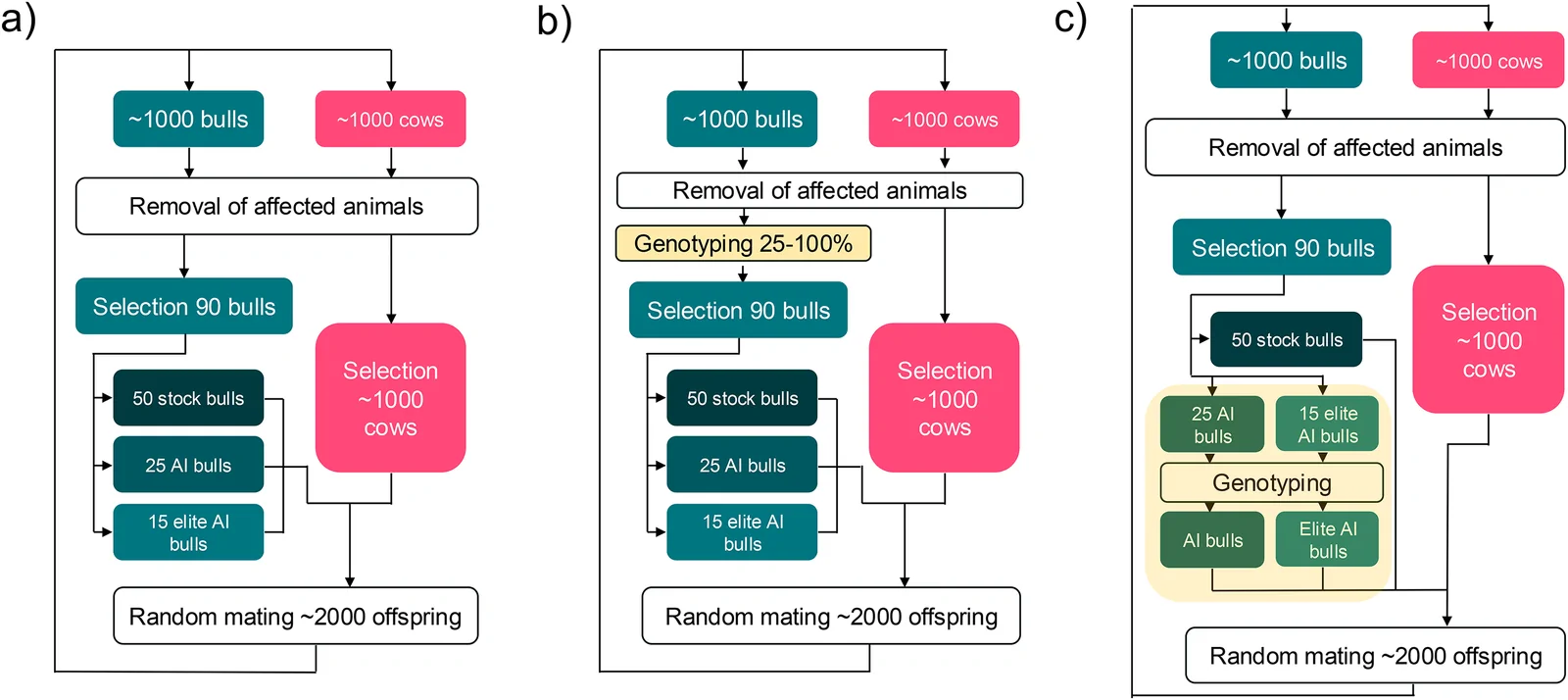
Overview of the scenarios, showing a) the baseline scenario without selection against the deleterious allele; b) pre-selection scenarios where genotyping and selection against the deleterious allele is performed on young males before selecting bulls; and c) post-selection scenarios where the genotyping and selection against the deleterious allele happens after selection of AI bulls.

Fig 2 provides a schematic overview of the scenarios.

### Evaluation of scenarios

In each replicate, the pedigree of all animals, the allele frequency of the deleterious allele, the frequency of affected animals, and the number of genetic tests were recorded.

Effective population size was calculated from pedigree data during generations 21–40 when alternative selection was implemented. Inbreeding coefficients were calculated using the inbreeding function within the GeneticsPed package [19]. The average inbreeding coefficient ${\overset{―}{F}}_{t}$ in each generation *t* and the standardised rate of inbreeding were calculated:


$$\begin{array}{c} \Delta F_{t}=\frac{{\overset{―}{F}}_{t}-{\overset{―}{F}}_{t-\text{1}}}{\text{1}-\;{\overset{―}{F}}_{t-\text{1}}} \end{array}$$

Then, the inbreeding effective population size [23] was calculated as:


$$\begin{array}{c} N_{e}=\frac{\text{1}}{\text{2}\;\overset{―}{\Delta F}} \end{array}$$

Here, $\overset{―}{\Delta F}$ is the average rate of inbreeding in generations 21–40. We used uncertainty intervals showing the showing the 5 and 95 percentiles to show the random variation over simulation replicates.

## Results

### Frequency of deleterious allele and affected animals

**Fig 3:**
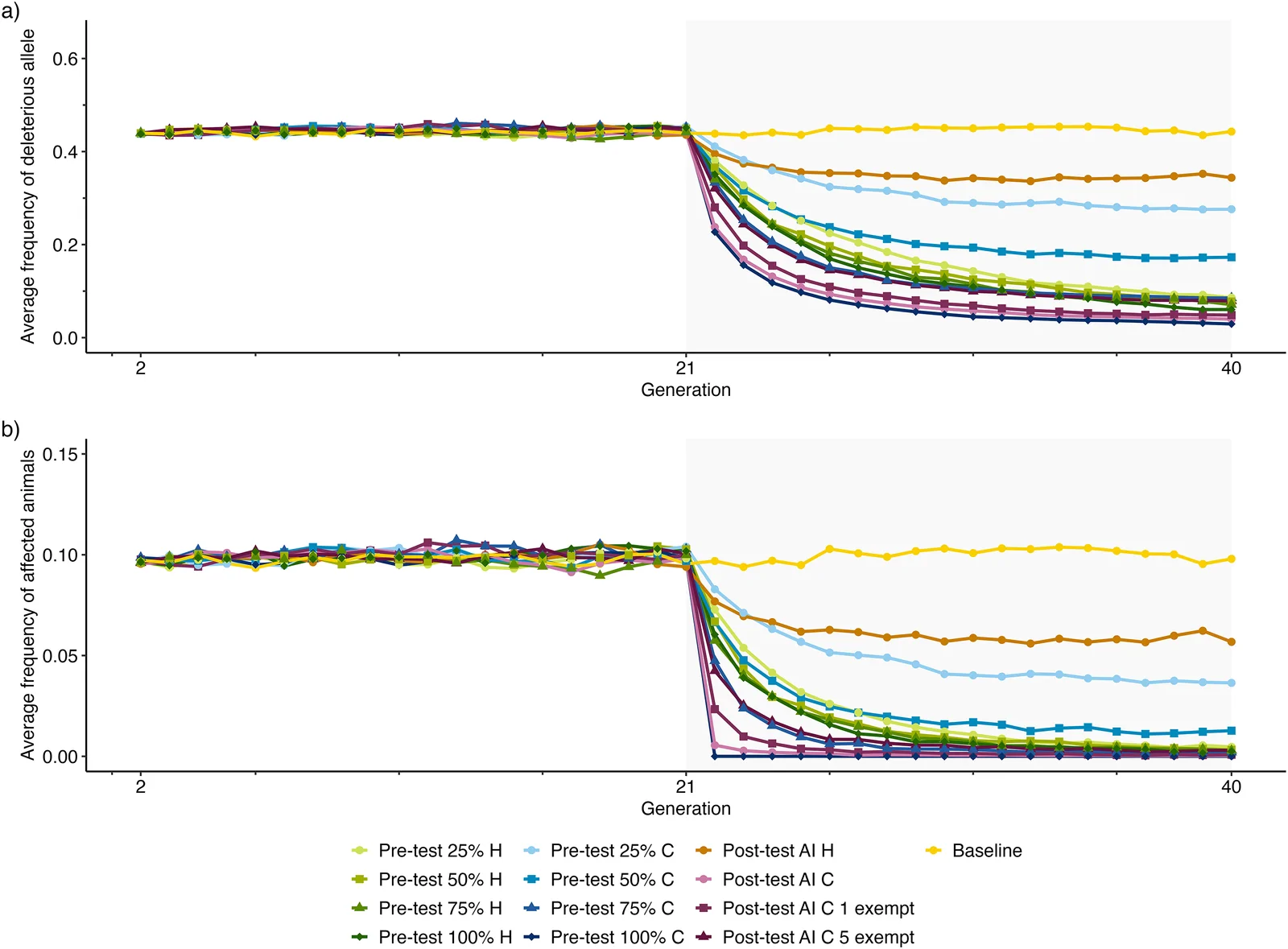
a) Average frequency of the deleterious allele across all simulated scenarios. b) Average frequency of affected animals across all simulated scenarios. The horizontal axis shows simulated generations. The grey background marks the period (generation 21-40) when alternative breeding strategies were implemented in all scenarios except the baseline scenario. Scenarios with “C” represent selection that excludes both carriers and homozygotes, while scenarios with “H” represent selection that excludes only homozygotes. In scenarios with 1 or 5 “exempt”, this number of carrier bulls were allowed among elite AI sires.

All simulated scenarios led to a reduction in average frequency of the deleterious allele, and the effect was larger when screening a larger proportion of the bulls, and when carriers rather than only homozygotes were removed from breeding. Fig 3 shows the trajectory of the average frequency of the deleterious allele and of affected animals across 40 generations for all simulated scenarios, and S2 Fig shows the frequency for each simulation replicate. Table 1 gives an overview of the results with frequencies and effective population sizes. The baseline scenario, mimicking a continuation of the current gonadal hypoplasia management strategy, resulted in little change on average, suggesting that this scenario was close to equilibrium. Testing all unaffected bulls to remove carriers of the deleterious allele was the scenario with the largest and most consistent reduction of the deleterious allele across all simulated scenarios and resulted in an allele frequency of 2.9% in the last generation. This scenario also eliminates production of affected offspring, since all bulls used are free from the allele. Selection against carriers among the AI sires was nearly as effective, with an allele frequency of 4.0% in the last generation. Selection against carriers among elite AI sires also resulted an immediate and drastic reduction in frequency of affected animals.

**Table 1:** Average frequency of deleterious allele and affected animals in generation 40, average effective population size and inbreeding rate, across generations 21 to 40 (with uncertainty intervals showing the 5 and 95 percentiles).

Scenario	Allele frequency	Affected frequency	Effective population size	Inbreeding rate (%)
Baseline	0.44 (0.39, 0.49)	0.098 (0.07, 0.13)	139 (119, 163)	0.364 (0.308, 0.419)
Pre-test 25% C	0.28 (0.22, 0.33)	0.036 (0.021, 0.055)	139 (110, 168)	0.366 (0.298, 0.454)
Pre-test 50% C	0.17 (0.12, 0.21)	0.013 (0.0035, 0.023)	139 (116, 172)	0.365 (0.291, 0.429)
Pre-test 75% C	0.085 (0.056, 0.12)	0.002 (0, 0.0055)	132 (109, 154)	0.384 (0.325, 0.458)
Pre-test 100% C	0.029 (0.014, 0.045)	0 (0, 0)	137 (118, 165)	0.371 (0.303, 0.424)
Pre-test 25% H	0.086 (0.028, 0.16)	0.0047 (0, 0.012)	136 (120, 163)	0.371 (0.307, 0.417)
Pre-test 50% H	0.078 (0.017, 0.16)	0.0032 (0, 0.0092)	140 (121, 157)	0.361 (0.319, 0.415)
Pre-test 75% H	0.071 (0.029, 0.13)	0.0028 (0, 0.0086)	137 (119, 157)	0.367 (0.319, 0.42)
Pre-test 100% H	0.06 (0.014, 0.12)	0.0025 (0, 0.008)	138 (118, 159)	0.366 (0.315, 0.424)
Post-test AI C	0.04 (0.024, 0.058)	3e-04 (0, 0.001)	94.5 (78.8, 111)	0.536 (0.452, 0.634)
Post-test AI C 1 exempt	0.049 (0.032, 0.075)	0.00081 (0, 0.0035)	73.2 (62.2, 84.5)	0.692 (0.592, 0.804)
Post-test AI C 5 exempt	0.079 (0.058, 0.11)	0.003 (0, 0.009)	94.2 (84.7, 107)	0.534 (0.466, 0.59)
Post-test AI H	0.34 (0.31, 0.39)	0.057 (0.043, 0.075)	126 (113, 149)	0.4 (0.335, 0.443)

Scenarios with “C” represent selection that excludes both carriers and homozygotes, while scenarios with “H” represent selection that excludes only homozygotes. In scenarios with 1 or 5 “exempt”, this number of carrier bulls were allowed among elite AI sires.

There was substantial variation in the frequency of the deleterious allele between replicates. The uncertainty interval for the average allele frequency in generation 40 across replicates was, bar one exception, wider in scenarios where only homozygous bulls were removed from breeding, indicating greater between-replicate variation in the final generation in these scenarios.

### Effective population size

None of the simulated scenarios involving genetic testing and removal of homozygous or carrier bulls prior to selection (pre-test) resulted in an average effective population size of less than 135 in generation 21–40. While there was variation between scenarios on average, there was no clear trend of decreasing N_e_ when the proportion of the males tested increased. In contrast, all three scenarios implementing selection against carriers among AI bulls and elite AI sires (post-test) resulted in substantial reductions in N_e_, below 100 when carriers and homozygotes were excluded. Selection against homozygotes among AI bulls did not have an equally detrimental effect on N_e_ as selection against carriers, resulting in an N_e_ on average below 130. The baseline scenario did not yield the highest average N_e_, illustrating the substantial random variation in realised effective population size.

### Number of genetic tests

Screening the population of bulls before selection (i.e., pre-test scenarios) requires many genetic tests, whereas screening AI bulls after selection (post-test) require few tests. Naturally, in all scenarios removing carrier or homozygous prior to selection, the number of genetic tests utilised per generation increased in tandem with the proportion of bulls tested. However, in pairwise comparisons of the sub-scenarios excluding either homozygotes or carriers, those excluding carriers consistently resulted in a higher number of genetic tests per generation compared to those with the same proportion tested excluding homozygotes (Table 2). Due to the design of the post-test selection scenarios, the number of tests used did not vary, with each of the four scenarios requiring exactly 40 genetic tests per generation. Although the last two sub-scenarios exempted either 1 or 5 bulls from the selection against carriers it is assumed that these bulls were still tested to obtain their genotype even if that information was not directly utilized to exclude them from breeding.

**Table 2:** Average number of genetic tests used per generation, (mean and uncertainty intervals showing the 5 and 95 percentiles), across generation 21-40.

Scenario	Gene tests performed per generation
Pre-test 25% C	239 (231, 242)
Pre-test 50% C	488 (470, 494)
Pre-test 75% C	740 (716, 747)
Pre-test 100% C	993 (992, 998)
Pre-test 25% H	245 (233, 248)
Pre-test 50% H	490 (469, 497)
Pre-test 75% H	737 (711, 746)
Pre-test 100% H	983 (945, 996)
Post-test AI C	40
Post-test AI C 1 exempt	40
Post-test AI C 5 exempt	40
Post-test AI H	40

Scenarios with “C” represent selection that excludes both carriers and homozygotes, while scenarios with “H” represent selection that excludes only homozygotes. In scenarios with 1 or 5 “exempt”, this number of carrier bulls were allowed among elite AI sires.

## Discussion

This study set out to evaluate breeding strategies aimed at reducing the frequency of gonadal hypoplasia and the associated Cs_29_ allele in Swedish Mountain cattle while preserving the breed’s genetic diversity. This case also serves an example of breeding strategies against genetic defects in vulnerable breeds, from which we can draw some general conclusions.

While the current policy of phenotypic selection against gonadal hypoplasia can be expected to maintain a relatively stable prevalence of Cs_29_ and the hypoplastic phenotype, the simulation results show that marker-assisted selection against the defect can reduce the frequency of the deleterious allele and of hypoplastic animals, but that there are trade-offs between reducing the defect, genetic diversity and cost. In the simulations, substantial reductions in the frequency of the deleterious allele, along with corresponding decreases in the number of affected animals, were rarely achieved without compromising effective population size or performing many genetic tests.

This trade-off is well illustrated by the scenario in which all unaffected bulls were tested to exclude carriers of the deleterious allele from breeding. Although such widespread testing is unlikely to be realistic in practice, it resulted in both the lowest average allele frequency and complete absence of affected animals, while maintaining effective population size. In contrast, subjecting only AI bulls to selection against carriers achieved a comparable reduction of the defect, but had the most detrimental impact on the genetic diversity, resulting in an average 31% reduction in effective population size. However, the scenario with complete testing of unaffected bulls required performing on average 25 times more genetic tests.

In the light of these results, we will discuss the aims of management of genetic defects, what effective population size should be targeted, what selection strategy that is most feasible in practice, assumptions and limitations of the analysis, and finally what general lessons can be drawn from this example.

### The aims of managing genetic defects

The trade-offs between reduction of the genetic defect, genetic diversity and cost raise the question whether the goal of the selection should be to avoid producing affected animals, to attempt to eliminate the deleterious allele from the population, or whether merely to reduce the risk of affected animals being born. Cole et al. [14] proposed that strategies aimed at avoiding homozygous offspring should be viewed as short-term solutions, whereas the reduction of the deleterious allele represents a more long-term approach. The main issue with hereditary gonadal hypoplasia is that it can cause sterility, which in turn, if a large enough proportion of the population is affected, may reduce the number of breeding animals and increasing the risk of inbreeding. Following this reasoning, it appears as if aiming to avoid homozygous offspring would be sufficient to render gonadal hypoplasia practically harmless by not allowing it to be expressed. However, if the deleterious allele remains in the population, measures such as genetic testing will continue to be necessary, for practical purposes indefinitely.

As for elimination of the allele, even the most intensive selection strategy in the current study failed to eliminate the deleterious allele from the population. Even if elimination of the allele is feasible, excluding all carriers of the deleterious allele from breeding may have detrimental impacts on both inbreeding as well as the genetic gain of other important traits [14,15]. A strategy that increased inbreeding rate and lowered effective population size could be counterproductive, as it would increase the risk that other previously unknown genetic defects rise to high frequency instead [24]. This depends on the prevalence of the deleterious allele in the population, if there are enough non-carriers to recruit replacement animals and if there are any genetic correlation to other traits under selection. Simulations by Leroy and Rognon [25] modelled on effectively small dog breeds found that when the initial frequency of the deleterious allele was lower (20%), the consequences for genetic variation (in their case measured by average coancestry) were minor, whereas when the frequency was high (50%), similar to the Mountain Cattle case, selection against carriers had a substantial detrimental effects on diversity. In species that produce many offspring, simulations by Rodríguesz-Ramilo et al. [26] suggest that management of deleterious alleles with maintained diversity is relatively easy, likely because segregation within families make it possible to find candidates that are otherwise genetically similar but without the defect. The problem is more challenging in a breed where there are few male candidates to choose from.

### The appropriate effective population size

Multiple recommendations have been proposed concerning threshold values for effective population size for a stable population. These typically include both short-term guidelines, spanning approximately five generations, and long-term minimum values for longer time scales. The short-term recommendations aim to prevent decreased fitness caused by inbreeding depression. A minimum effective population size of 50, corresponding to an inbreeding rate of 1%, was long regarded as the standard recommendation to prevent inbreeding depression in the short term [27]. However, Frankham et al. [28] instead argued that the effective population size should not be less than 100, corresponding to an inbreeding rate of 0.5%. The same authors also proposed an increase of the long-term recommendation from an effective population size of above 500 to instead above 1000. The goal of the long-term recommendation is to ensure adequate genetic variation to adapt to potential future changes and challenges. Hardly any livestock breeds, and none of the simulated scenarios, meet this second criterion [29].

The molecular estimates [22] that the simulation were based on suggest that the Swedish Mountain cattle breed has an effective population size of around 130, falling above the short-term recommendation but far below the long-term recommendation. In the light of this, we would argue that an intervention against a genetic defect should, ideally, not lower effective population size at all, and certainly not make it drop below 100. Most scenarios did reach an average effective population size exceeding 100 (0.5% inbreeding rate), meeting the short-term recommendation. However, the scenarios that reduced effective population size below 100, due to reducing the number of AI bulls used, cannot be recommended.

Furthermore, there was considerable variation in the realised effective population size across all scenarios. Such variability underscores the importance of continuous monitoring of genetic diversity, regardless of which management strategy is implemented to avoid unintended erosion of genetic variation.

### Practical genotyping and selection strategies

The results also show that the timing of the genetic testing matters to both diversity and cost. If we assume a testing cost of 1000 SEK per test, which is in line with similar genotyping tests, post-selection tests of AI bulls cost 40,000 SEK per generation or around 5000 SEK per year. Testing of the unaffected bull population prior to selection would lead to the greatest reduction in the defect with no loss of effective population size, but this strategy would cost 993,000 SEK per generation. Even the most limited pre-test scenario that was simulated would cost 239,000 SEK per generation. Therefore, the most promising option seems to be to develop a strategy to genetically test a pre-selected subset of candidate AI bulls, maintaining the number of AI bulls while genetically testing a relatively small number of animals. If promising bull calves can be tested at an early age, carriers can be removed early, before a large investment is made into raising them. When DNA tests from earlier generations are available, segregation analysis can also be used to reduce the testing burden by identifying offspring that need not be tested because they cannot have inherited the allele [30].

Given that complete elimination of the genetic defect is unlikely in the foreseeable future, we might ask how large a reduction in frequency is needed to be worthwhile. The simulations show that even in the case where 25% of unaffected bulls were genetically tested, the frequency of affected animals decreased below 5% on average after a few generations. Because the frequency of affected animals declines quadratically with allele frequency, and is approximately halved because of incomplete penetrance, even a relatively small reduction in frequency matters. If a test was made available to farmers, if even a small proportion would genotype their animals for selection against carriers, this would likely reduce the number of affected animals with no detrimental effect on the effective population size. Farmers who use the test are likely to also use it for selecting recruitment heifers, meaning that the assumption of selection only on the male side is conservative. In addition, genotypes gathered during validation and testing of the marker may serve to increase the proportion of the population screened during the initial phase, to no cost to the farmers and breed society.

Another management option is planned mate allocation where genetic information at the Cs_29_ locus is used to avoid at-risk matings while still using carriers of the Cs_29_ allele in breeding. Several such methods exist that can balance selection against defects with controlling inbreeding rate and avoid production of affected offspring [31–34]. They assume varying degree of control of the breeding scheme. For example, Cole [31] evaluated a method for selecting service sires for the cows in a herd based on breeding values penalised by inbreeding and risk of expression of recessive deleterious alleles. However, for schemes that require the genotypes of each mating pair, widespread application in local breeds seems far-fetched. Sonesson et al. [34] on the other hand, assumed no control over the females, optimising the contributions of males based on average coancestry and genotypes. They did, however, assume control of the contributions of the selected sires, implying centralised control over sires used. This degree of central mate planning appears unlikely in the context where breeding decisions are made by farmers.

Genotyping and publishing of the carrier status of AI bulls would provide farmers with the information to select non-carrier bulls if they are not willing to genotype their cows, and safely use of carrier bulls on cows that are tested free of the defect. A note of caution is warranted, as this strategy was not simulated. There is a risk that publishing carrier status of bulls may shift the distribution of the contributions of AI bulls similar to the post-selection scenarios in this simulation, which may affect the effective population size, e.g., by changing the variance of offspring numbers [35] or the average relatedness of the bulls used to the population.

Because hypoplasia occasionally causes sterility, and more often likely leads to subfertility, there are clearly economic costs to the defect. However, because the milk production of this breed is only recorded for a small part of the population, there is not data to estimate economic losses due to lower fertility from gonadal hypoplasia. This makes it difficult to assess the economic value of genotyping, and impairs the use of mate allocation methods that penalise animals based on the cost of the genetic defect, e.g., [31,32].

### Assumptions and limitations

This section will identify and discuss methodological limitations that have not yet been addressed.

Throughout the paper, we have assumed that the Cs_29_ allele was strongly associated with gonadal hypoplasia and that there is no genotyping error. This could be either because Cs_29_ is the causative variant or that the variants are in strong linkage disequilibrium. An association study from Northern Finncattle, a breed closely related to Swedish Mountain cattle, supports the association between Cs_29_ and hypoplasia [5]. However, the association has not been validated in Swedish Mountain cattle, and that needs to be done before recommending selection based on Cs_29_ genotypes. The arguments in this paper apply equally to other potential genetic markers for gonadal hypoplasia. Furthermore, we have assumed that the dominance relationships and penetrance are unchanged from the estimates of Eriksson et al. [1].

The breeding structure of the simulated population is simplified in several ways. We assumed discrete generations and a constant breeding structure, which is not realistic but greatly simplifies modelling. There were several differences between the simulated baseline selection and the current breeding strategy and management of gonadal hypoplasia. The number of bulls in the three fecundity groups and the number of offspring assigned to each bull likely do not exactly reflect the actual population. However, without access to accurate data on the use of AI bulls or detailed records of the number of calves born from these bulls it was difficult to construct a more realistic scenario. In addition, we placed no constraint on the number of offspring assigned to each dam, meaning that some dams randomly produced more offspring than would be biologically possible during a five-year period. Similarly, there were no restrictions on relatedness when assigning mating pairs. As a result, individuals that under real circumstances would not have been allowed to breed due to their level of kinship were still able to do so in the simulations. Thus, the breeding structure and in the simulations was not entirely representative of the actual population. However, collectively they yielded an effective population size close to that of the real population indicates that they, providing a reasonable baseline for comparing different scenarios.

The simulated scenarios of selection against carriers among AI bulls represent the most pessimistic case where AI bulls found to be carriers were excluded and none of them could be replaced. Further, the relative proportions of matings contributed by the high and medium fecundity AI sires were kept constant after exclusion of carriers, meaning that popular bulls received a larger number of matings than in the baseline scenario. This is a pessimistic design, but we believe also realistic, in the sense that popular bulls remain popular when marker-assisted selection against carriers is implemented. However, if excluding carrier bulls would lead to a more even distribution of offspring between bulls, the consequences of effective population size may not be as severe. To further explore this, more realistic models of the distributions of offspring within the population would be needed.

We assumed that there was positive selection for the colour-sided phenotype, which has been historically the case, but is no longer part of the breeding goal [36]. For simplicity, we assumed that this preference was only driven by males, which is likely not entirely accurate. However, that fact that both frequencies of gonadal hypoplasia and the associated Cs_29_ allele remain high, at a similar frequency in the 1950s [9] and in the 2020s [12] despite the ongoing selection against the defect suggests the presence of a balancing selection, most likely in the form of a preference for colour-sidedness. As for the strength of this preference, the simulations suggest that it should be close to $s_{\text{1}}\approx \text{0}.\text{3}$, otherwise the equilibrium frequency would diverge from the observed allele frequency in the breed. Coefficients of positive selection of 0.4 or 0.2 would lead to markedly higher or lower allele frequencies, respectively.

## Conclusions

In conclusion, we can separate practical management strategies into two categories: voluntary testing by farmers, which can eliminate expression of hypoplasia on their farms and contribute to reduction of allele frequency in the population at large, and coordinated efforts targeting recruitment of AI bulls, which need to be careful not to reduce the number of bulls used and thus harm effective population size. Our analysis suggests that it is feasible to reduce the frequency of the gonadal hypoplasia defect by marker-assisted selection, but that elimination is unlikely within the foreseeable future, and that selection that reduces the number of AI bulls would harm the genetic diversity of the breed and should be avoided. These results suggest that efforts to genotype and phenotype cattle for validation and testing of the marker in Swedish Mountain cattle is warranted.

## Supporting information

S1 Fig Tests of different values for the coefficient of positive selection s_1_ and the resulting equilibrium frequency. Each line is the average of 10 replicates. The horizontal dashed line shows the observed allele frequency of 0.44.
(PNG)

S2 Fig Frequency of the deleterious allele across all simulated scenarios, showing every replicate. Each line represents a replicate. The panels represent different scenarios.
(PNG)
